# Conversion of Cellobiose to Formic Acid as a Biomass‐Derived Renewable Hydrogen Source Using Solid Base Catalysts

**DOI:** 10.1002/open.202400079

**Published:** 2024-10-07

**Authors:** Ikuto Yoshiki, Atsushi Takagaki, Jun Tae Song, Motonori Watanabe, Tatsumi Ishihara

**Affiliations:** ^1^ Division of Materials Science and Chemical Engineering Faculty of Engineering Yokohama National University 79-5 Tokiwadai, Hodogaya-ku Yokohama Kanagawa 240-8501 Japan; ^2^ Department of Applied Chemistry Faculty of Engineering Kyushu University 744 Motooka, Nishi-ku Fukuoka 819-0395 Japan; ^3^ International Institute for Carbon-Neutral Energy Research (WPI−I2CNER) Kyushu University 744 Motooka, Nishi-ku Fukuoka 819-0395 Japan

**Keywords:** Cellobiose, formic acid, hydrogen peroxide, calcium oxide, solid base

## Abstract

Formic acid is considered a promising hydrogen carrier. Biomass‐derived formic acid can be obtained by oxidative decomposition of sugars. This study explored the production of formic acid from cellobiose, a disaccharide consisting of d‐glucose linked by β‐glycosidic bonds using heterogeneous catalysts under mild reaction conditions. The use of alkaline earth metal oxide solid base catalysts like CaO and MgO in the presence of hydrogen peroxide could afford formic acid from cellobiose at 343 K. While CaO gave 14 % yield of formic acid, the oxide itself was converted to a harmful metal peroxide, CaO_2_ after the reaction. In contrast, MgO could produce formic acid without the formation of the metal peroxide. The difficulty in selectively synthesizing formic acid from cellobiose using these solid base catalysts was due to the poor conversion of cellobiose to glucose. Using a combination of solid acid and base catalysts, a high formic acid yield of 33 % was obtained under mild reaction conditions due to the quantitative hydrolysis of cellobiose to glucose by a solid acid followed by the selective decomposition of glucose to formic acid by a solid base.

## Graphical abstract

The oxidative conversion of the disaccharide cellobiose to formic acid was carried out. Formic acid was produced in the presence of hydrogen peroxide using solid base catalysts such as CaO and MgO, but the low yield was low. A two‐step one pot synthesis using solid acid and base catalysts improved the formic acid yield under mild reaction conditions.

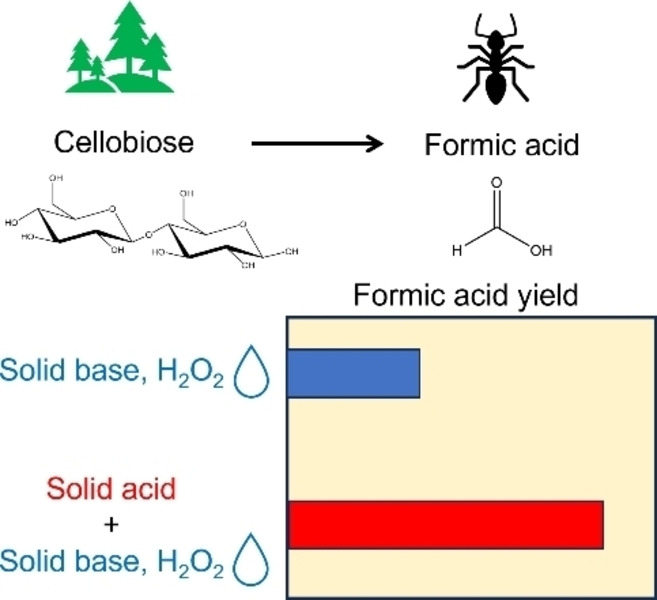

## Introduction

Production of formic acid has attracted attention because it has remarkable properties including high hydrogen content (4.4 %), low toxicity, and nonflammability, thus it is considered as a representative storage material for hydrogen.^[1]^ From formic acid, highly efficient hydrogen generation is nowadays possible under mild reaction conditions by using homogeneous^[2]^ and heterogeneous^[3]^ catalysts. Although there is widespread research on the synthesis of formic acid through catalytic hydrogenation^[4]^ and electrochemical reduction^[5]^ of CO_2_, it can be also produced from lignocellulosic biomass by oxidative conversion.^[6]^ Lignocellulosic biomass originally grows by absorbing CO_2_ and water through photosynthesis. Thus, hydrogen obtained via decomposition of biomass‐derived formic acid is renewable and environmentally friendly.

**Scheme 1:**
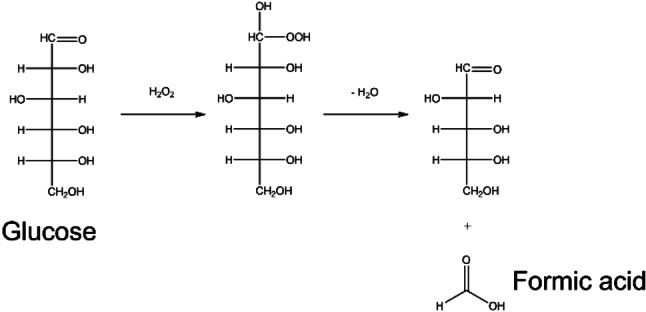
A possible reaction pathway for oxidative formic acid production from d‐glucose using base catalyst in the presence of H_2_O_2_.

In our previous study, calcium oxide, an abundant alkaline earth metal oxide solid base, was found to produce formic acid from monosaccharides including glucose, fructose, and xylose when H_2_O_2_ was used as an oxidant.^[7]^ High yields of formic acid of 50 % and 66 % were obtained from glucose and xylose, respectively at 343 K for 0.5 h. The base‐catalyzed oxidation to formic acid is supposed to proceed via the formation of an OOH^−^ species from base‐activated H_2_O_2_, followed by its attack on the aldehydic carbon atom of the acyclic aldoses such as glucose, resulting in C−C bond cleavage with the formation of formic acid (Scheme 1).[^8^, ^9^] This reaction pathway is desirable for selective formic acid formation because the sugar remains in aldose form during decreasing one carbon. There are other concomitant reaction pathways which are isomerization of glucose to fructose and the retroaldol reaction from hexose to triose. From fructose as a ketose, not only formic acid but also glycolic acid could be formed, which was hard to convert into formic acid in the system.

In the present study, we have examined the study of formic acid synthesis from cellobiose, a disaccharide consisting of glucose connected with β‐glycosidic bonds by using solid base catalysts in the presence of H_2_O_2_ as an oxidant under mild reaction conditions. Alkaline earth metal oxides like CaO and MgO were found to produce formic acid directly from cellobiose. Furthermore, a combination of solid acid and base catalyst was more effective for improving formic acid yield.

Efficient formic acid synthesis from cellobiose has been reported using homogeneous base catalysts.[^10^, ^11^] Concentrated NaOH (1.5 m) at 423 K^[10]^ and homogeneous LiOH with 200 % H_2_O_2_ ^[11]^ gave approximately 80 % and 73.7 % yield of formic acid, respectively. Ionic‐liquid‐based polyoxometalate hybrids were also found to catalyze the conversion of cellobiose to formic acid and levulinic acid, affording 26.1 % yield of formic acid under 3 MPa of oxygen.^[12]^ Our present study demonstrated that formic acid was selectively produced by using abundant solid base catalysts and a conventional solid acid under mild reaction conditions.

## Results and Discussion

### Conversion of Cellobiose to Formic Acid over CaO

**Figure 1:**
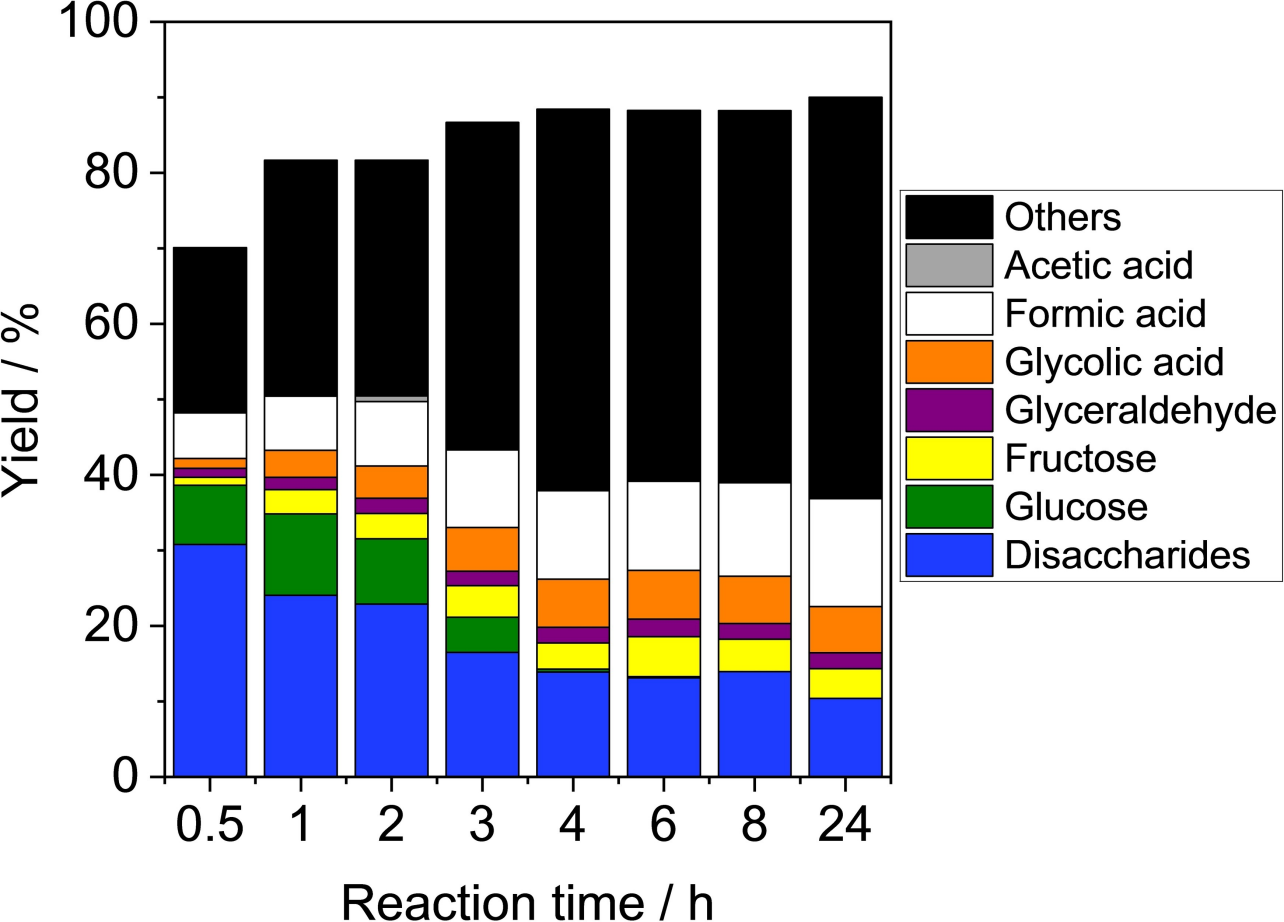
Formic acid formation from cellobiose over CaO catalysts using H_2_O_2_ as an oxidant. Reaction conditions: Cellobiose (0.17 g (0.50 mmol)), CaO (0.17 g), H_2_O_2_ (0.61 mL (6.0 mmol)), water (5 mL), 343 K.

The first trial of cellobiose conversion was conducted by using a CaO catalyst because it showed the highest activity for glucose conversion to formic acid as mentioned above.^[7]^ Figure 1 shows the results of cellobiose conversion over CaO with different reaction time. Formic acid was formed along with glucose and disaccharides in a 0.5 h reaction at 343 K. As reaction time increased, the yield of formic acid increased whereas the yields of glucose and possible disaccharides, including base‐catalyzed isomerization products such as cellobiulose and glucosyl‐mannose,^[13]^ decreased. Formic acid yield was 6 % after 0.5 h of reaction but improved to 9 % after 2 h and 12 % after 4 h. At that time, the yield of glucose was negligible (0.4 %), and instead, fructose was produced by isomerization of glucose, and simultaneously, glycolic acid and glyceraldehyde were formed in large amounts via retroaldol reaction of fructose. Furthermore, when the reaction time was extended to 24 h, the yield of formic acid increased to 14 %. As reaction time increased, others′ yield became larger, accounting for roughly half of the total. This was much different from our previous results where the monosaccharide glucose was tested as the reactant.^[7]^ The increase in others’ yield corresponded to a decrease in disaccharides yield, suggesting that although some of the cellobiose is successfully hydrolyzed to the monosaccharide glucose, the remainder isomerized as disaccharides, which over time converted to other unknown compounds that could not be analyzed by HPLC.

**Figure 2:**
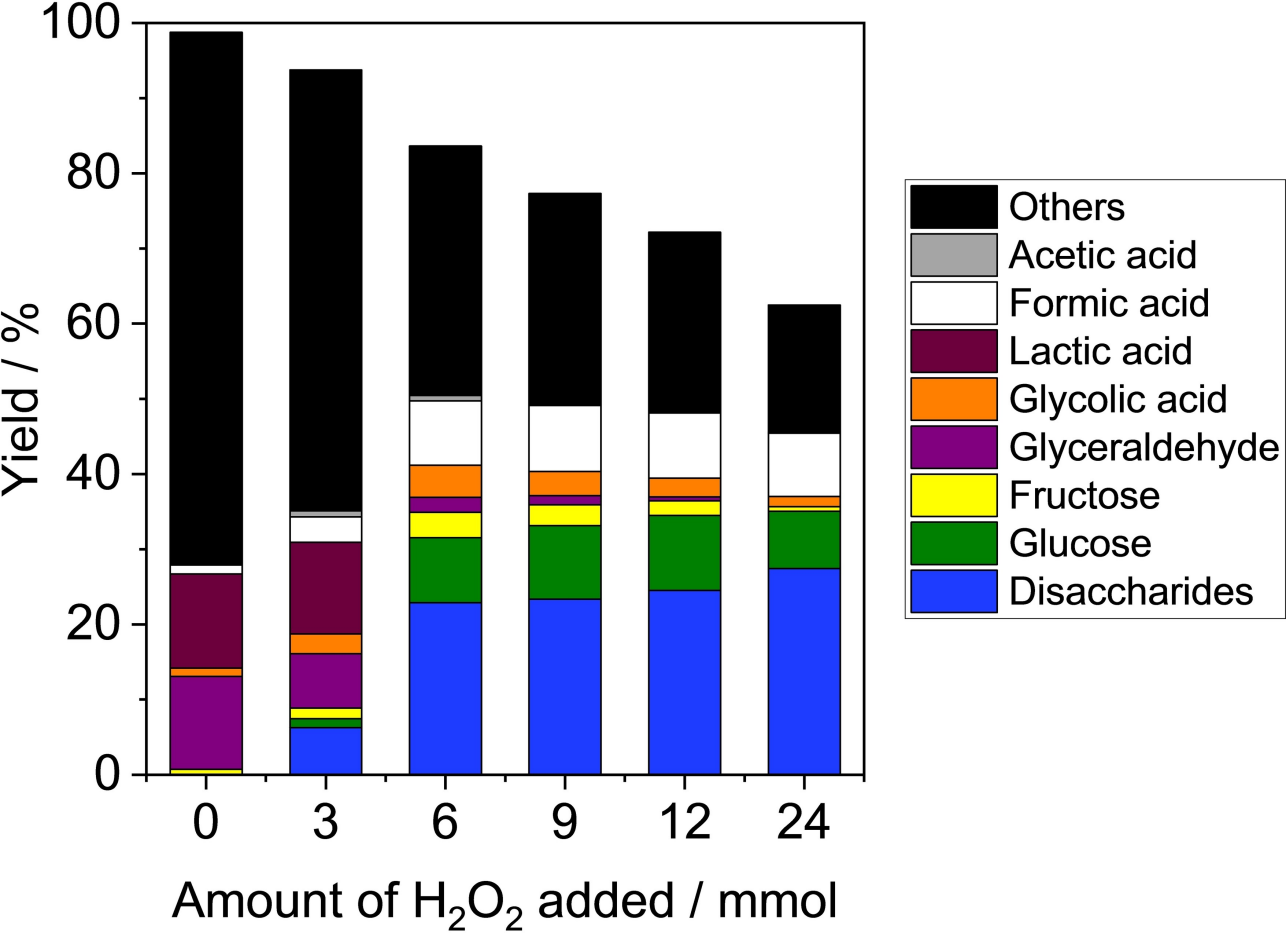
Effect of amount of H_2_O_2_ added on the formic acid formation from cellobiose. Reaction conditions: Cellobiose (0.17 g (0.50 mmol)), CaO (0.17 g), H_2_O_2_ (0–24 mmol)), water (5 mL), 343 K, 2 h.

Since a desirable reaction for the production from cellobiose by using H_2_O_2_ is expressed as C_12_H_22_O_11_+12 H_2_O_2_→12 HCOOH+11 H_2_O, in this study, the reaction was typically carried out using 12 equivalents of hydrogen peroxide (6 mmol) to cellobiose (0.5 mmol). Figure 2 shows the effect of H_2_O_2_ amount added to the reaction. In the absence of H_2_O_2_, no formation of formic acid was observed. Instead, a moderate yield of lactic acid (12 %) was obtained, which is due to other sequential base‐catalyzed reactions including the retroaldol reaction of hexoses to trioses, dehydration to pyruvaldehyde as an intermediate followed by transformation to lactic acid.^[14]^ The yield of formic acid was 9 % with 6 mmol of H_2_O_2_. When the amount of H_2_O_2_ was increased, the yield of formic acid decreased slightly with the decrease of cellobiose conversion. Therefore, it was found that the excess amounts of H_2_O_2_ were not necessary for improving the formic acid yield.

**Figure 3:**
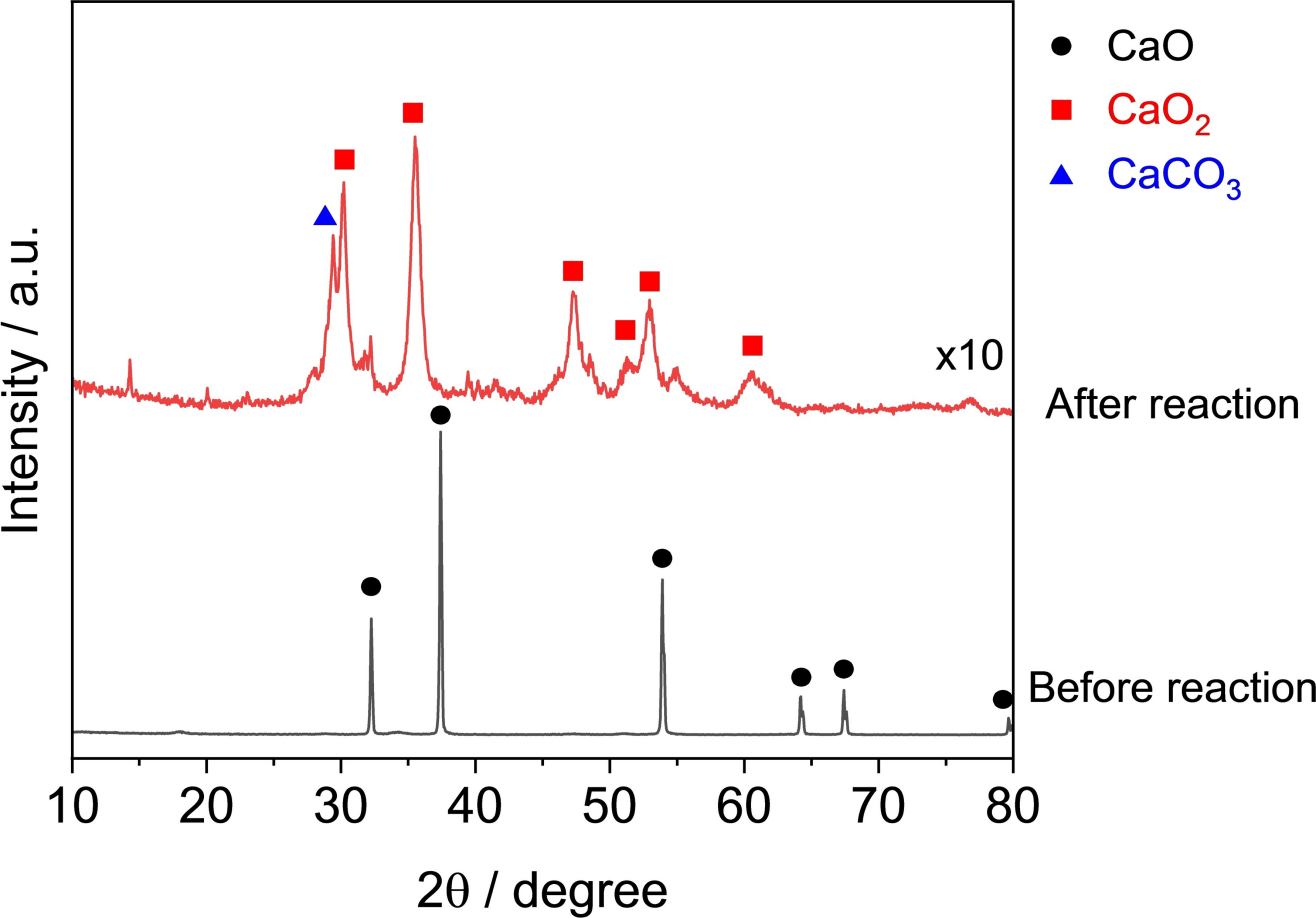
XRD patterns of CaO before and after reaction.

Change in the crystal structure of CaO before and after reaction was assessed by using XRD measurement. Figure 3 shows the results of XRD patterns. It was shown that the used sample was completely converted to calcium peroxide, CaO_2_, indicating that H_2_O_2_ was consumed not only by the oxidation of sugars but also by the reaction with CaO as the undesirable reaction, CaO+H_2_O_2_→CaO_2_+H_2_O. While the formed CaO_2_ could exhibit the activity for cellobiose decomposition to formic acid, the yield of formic acid was 5 %, much lower than that of CaO. From these results, CaO was found to be an inappropriate material for the conversion of cellobiose to formic acid because of its moderate activity and undesirable formation of CaO_2_ by consumption of H_2_O_2_.

### Conversion of Cellobiose to Formic Acid Using Alkaline Earth Metal Oxides

**Figure 4:**
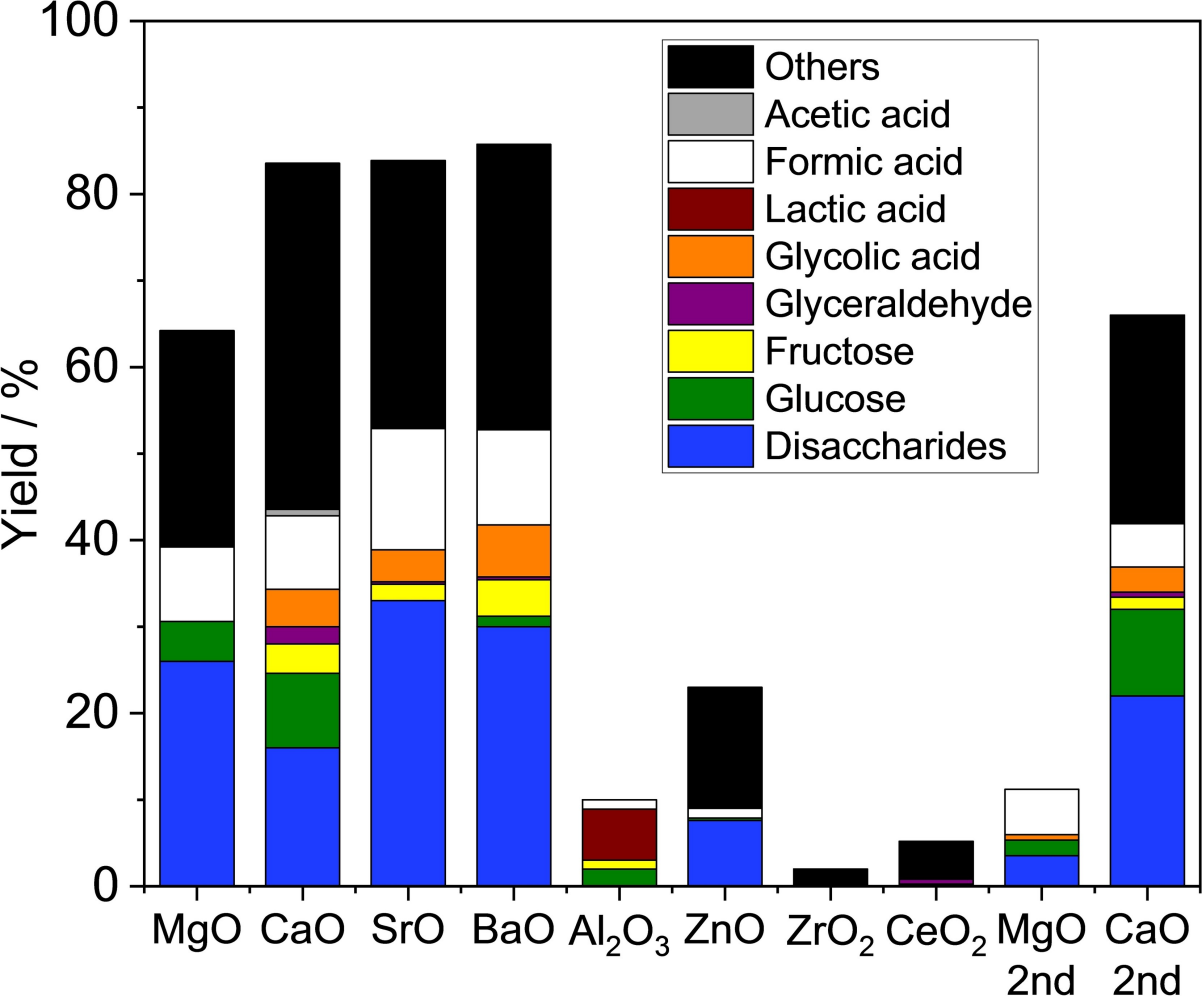
Formic acid production from cellobiose using a variety of metal oxides. Reaction conditions: Cellobiose (0.17 g (0.50 mmol)), metal oxide (0.17 g), H_2_O_2_ (6 mmol)), water (5 mL), 343 K, 2 h.

**Figure 5:**
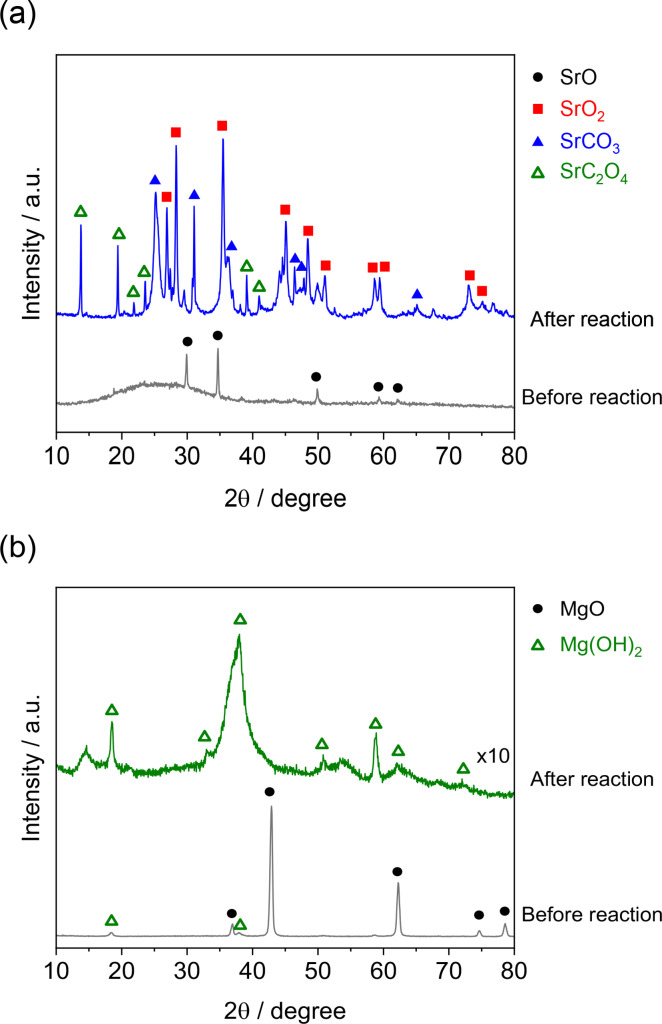
XRD patterns of (a) SrO and (b) MgO before and after reaction

Next, possible metal oxides that produce formic acid but avoid the formation of peroxide compounds like CaO_2_ during the reaction were explored. Figure 4 shows the results of formic acid production from cellobiose using a variety of metal oxides. Similar to the case of formic acid production from glucose,^[7]^ alkaline earth metal oxides were found to afford formic acid. The yield of formic acid was 9 %, 9 %, 14 %, and 11 % over MgO, CaO, SrO, and BaO, respectively. Although SrO gave the highest yield of formic acid among the samples tested, it was also converted to its peroxide, SrO_2_ as shown in Figure 5(a). In contrast, the metal peroxide was not formed for MgO as depicted in Figure 5(b). Furthermore, MgO did not produce glycolic acid which is advantageous for improving the selectivity of formic acid. In our previous study of formic acid production from glucose, glycolic acid was considered an unacceptable final byproduct because glycolic acid remained unchanged during the reaction and was hard to convert into formic acid.^[7]^ MgO and CaO were reused after decantation and drying at 353 K overnight. The formic acid yields of the reused MgO and CaO were 5.2 % and 5.0 %, respectively, though cellobiose conversion decreased significantly due to the change in the crystal structure (Figure 4). Experiments with *tert*‐butyl hydroperoxide (TBHP) as another oxidant were also tested. Table 1 lists the results of cellobiose conversion over MgO and CaO in the presence of H_2_O_2_ or TBHP. Similar activity was observed with MgO and CaO when H_2_O_2_ was used, but the activity was very different when TBHP was used; the conversion and formic acid yield were low when MgO was used in the presence of TBHP, but the conversion and formic acid yield were very high when CaO was used. The suppression of disaccharide and glucose formation and the large difference in activity depending on the type of oxide suggests a different reaction mechanism than when H_2_O_2_ is used. Although TBHP, an organic peroxide, has excellent oxidation ability, this study focused on selective oxidation with H_2_O_2_ as an environmentally friendly oxidant.

**Table 1:** Cellobiose decomposition over MgO and CaO in the presence of H_2_O_2_ or TBHP^*a*^

Catalyst	Oxidant	Conv. /%	Yield /%							
			DS	GLU	FRU	GLA	GA	FA	AA	Others
MgO	H_2_O_2_	64	26	4.6	0	0	0	8.6	0	25
MgO	TBHP	11	0	3.7	0.2	0.3	0.4	2.0	0	4.4
CaO	H_2_O_2_	84	16	8.6	0	0	0	8.5	0.8	40
CaO	TBHP	99	2.4	0.2	0	3.5	5.1	56	0	32

^*a*^ *Reaction conditions*: Cellobiose (0.17 g (0.50 mmol)), metal oxide (0.17 g), oxidant (6 mmol)), water (5 mL), 343 K, 2 h. ^*b*^ DS: disaccharides, GLU: glucose, FRU: fructose, GLA: Glyceraldehyde, GA: Glycolic acid, FA: Formic acid, AA: Acetic acid.

### Two‐Step, One‐Pot Synthesis of Formic Acid from Cellobiose Using Solid Acid and Base Catalysts

Although MgO could selectively produce formic acid without the formation of its peroxide, the yield of formic acid was still low as the case using other alkaline earth metal oxides. This is mainly due to the lack of ability for hydrolysis of cellobiose to glucose. Solid acid catalysts are known to efficiently catalyze the hydrolysis of saccharides^[15]^ and combining solid acid and base catalyst in a single reactor allows each catalytic reaction to proceed without neutralization.^[16]^ Therefore, the hydrolysis of cellobiose was first carried out with a solid acid catalyst, followed by the use of a solid base catalyst and hydrogen peroxide to obtain formic acid from glucose.

**Scheme 2:**
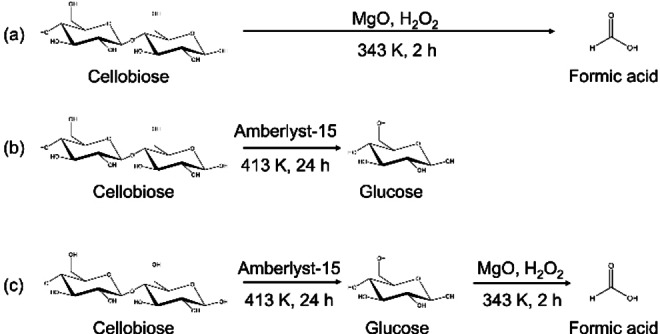
(a) Direct decomposition of cellobiose using a solid base catalyst, MgO in the presence of H_2_O_2_ as an oxidant, (b) hydrolysis of cellobiose using a solid acid catalyst, Amberlyst‐15, (c) production of formic acid via acid‐catalyzed hydrolysis followed by base‐catalyzed oxidative decomposition of glucose by using solid acid and base catalyst.

**Figure 6:**
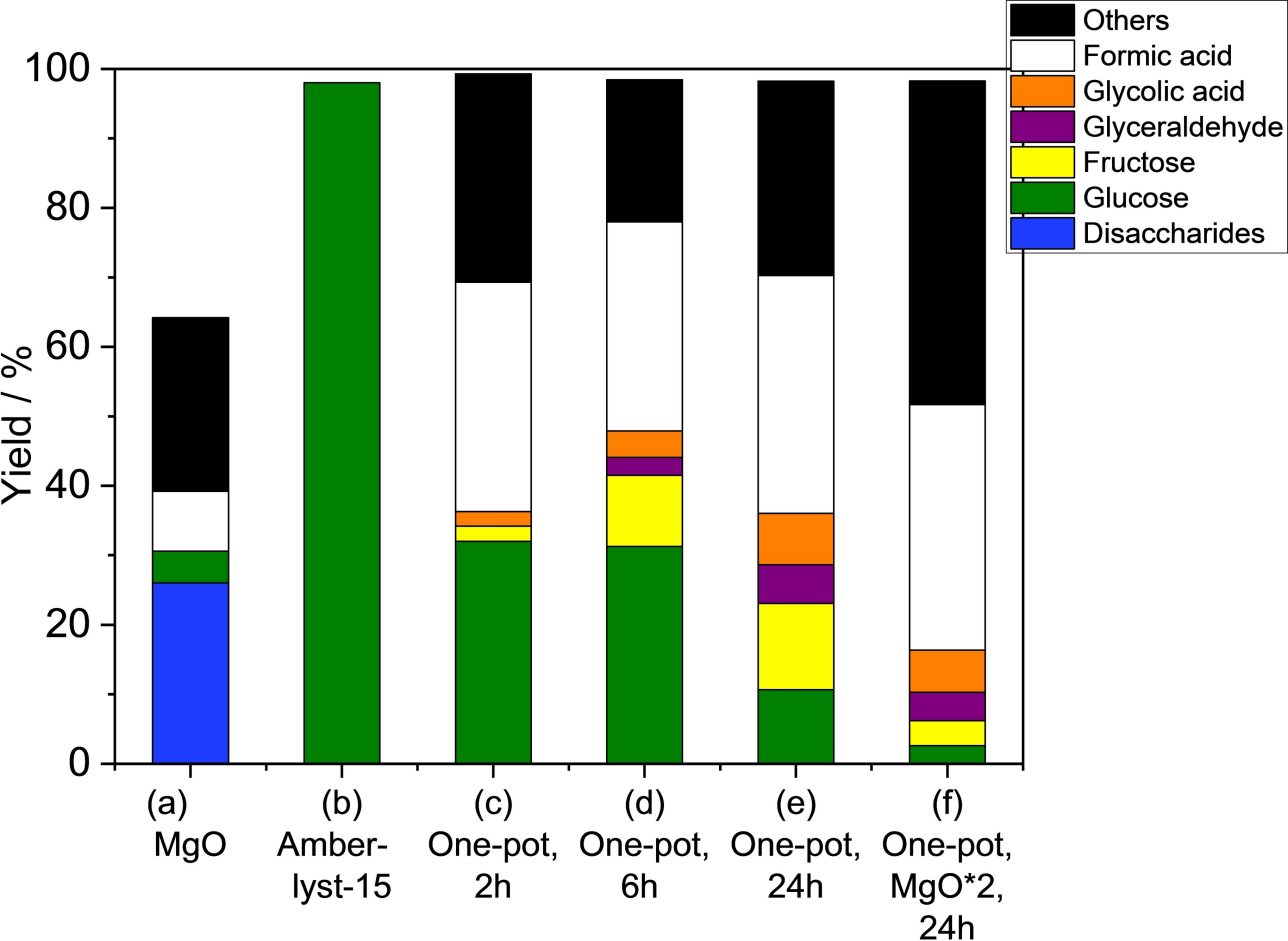
(a) Formic acid production from cellobiose using MgO. Reaction conditions: Cellobiose (0.17 g), MgO (0.17 g), H_2_O_2_ (6 mmol)), water (5 mL), 343 K, 2 h. (b) Hydrolysis of cellobiose using Amberlyst‐15. Reaction conditions: Cellobiose (0.18 g), Amberlyst‐15 (0.36 g), water (5 mL), 413 K, 24 h. (c–f) Two‐step, one‐pot reaction. Hydrolysis of cellobiose using Amerlyst‐15 at 413 K for 24 h followed by oxidative decomposition of glucose by addition of MgO (0.17 g (c–e) or 0.34 g (fI) and H_2_O_2_ (6 mmol) at 343 K for 2 h (c), 6 h (d), and 24 h (e,f).

Scheme 2 and Figure 6 show the reaction scheme and results of each reaction. As mentioned above, the single use of MgO as a solid base catalyst for direct conversion of cellobiose results in a low yield of formic acid (Scheme 2(a) and Figure 6(a)). Single‐use of Amberlyst‐15, a styrene‐based sulfonic acid resin as a representative solid acid catalyst, afforded almost full conversion of cellobiose (98 %) with excellent selectivity to glucose at 413 K for 24 h (Scheme 2(b) and Figure 6(b)). After the reaction of cellobiose using Amberlyst‐15 in water to produce glucose, MgO and hydrogen peroxide were simultaneously added to the reactor vessel without separation of solid acid ion‐exchange resin (Scheme 2(c) and Figure 6(c)), resulting in high‐yield of formic acid (33 %). Thus, two‐step, one‐pot synthesis using solid acid and base catalysts was found to be an effective way to afford a high yield of formic acid from cellobiose.

Since glucose remained in the 2 h reaction, the reaction time was extended to 6 and 24 h (Figure 6(d)(e)). Glucose decreased with time. After 24 h, glucose decreased to 11 % yield, while the yield of formic acid increased only slightly (34 %). The reaction was also carried out with double the amount of MgO for 24 h (Figure 6(f)). In this case, most of glucose was converted and the yield of formic acid was 35 %. Although the extended time decreased the yield of glucose, it did not significantly improve the yield of formic acid, which was mainly due to isomerization of glucose to fructose and retroaldol reaction to triose, and subsequent transformation to byproducts (others).

## Conclusions

The conversion of cellobiose to formic acid was examined using alkaline earth metal oxides as solid base catalysts in the presence of H_2_O_2_ as an oxidant. While CaO could give a 14 % yield of formic acid at 343 K for 24 h reaction, the oxide itself was converted to a harmful metal peroxide, CaO_2_. In contrast, the peroxide formation was suppressed for MgO which could selectively afford formic acid without the formation of glycolic acid. A combination of Amberlyst‐15 as a solid acid catalyst for the hydrolysis of cellobiose to glucose and MgO as a solid base catalyst for the decomposition of glucose to formic acid using H_2_O_2_ allowed high formic acid yield of 33 % under mild reaction conditions.

## Experimental Section

### Chemicals

d(+)‐Cellobiose (Wako), d(+)‐maltose monohydrate (98 %, Wako), d(+)‐glucose (98 %, Kishida), d(−)‐fructose (99 %, Wako), dl‐glyceraldehyde (>90 %, Sigma‐Aldrich), glycolic acid (97 %, Wako), lactic acid (85–92 %, Nacalai‐Tesque), acetic acid (99.7 %, Nacalai‐Tesque), formic acid (98 %, Wako), hydrogen peroxide (30 %, Wako), *tert*‐butyl hydroperoxide (70 % in water, TCI) and benzoic acid (99.5 %, Wako) were used for the reactions and the analysis. Calcium oxide (CaO, 99.9 %, Wako), magnesium oxide (MgO, 99.9 %, Wako), and other metal oxides, SrO (95 %, Nacalai‐Tesque), BaO (97 %, Sigma‐Aldrich), Al_2_O_3_ (Wako), ZnO (99.9 %, Wako), ZrO_2_ (98 %, Wako), and CeO_2_ (99.9 %, Rare Metallic) were used as catalysts. An ion‐exchange resin, Amberlyst‐15 (Sigma‐Aldrich) was also used as a solid acid catalyst.

### Characterization

X‐ray diffraction (XRD) analysis was conducted to determine the crystal structure of the catalysts. The diffractometer (RINT‐2500HLR+, Rigaku) was operated with Cu Kα radiation generated at 40 kV and 80 mA. Scans were obtained at a speed of 5° min^−1^ with a step width of 0.05° for 2θ values from 10 to 80°.

### Catalytic Reaction

The reaction was conducted using a glass reactor vessel with pressure resistance (ACE glass). Typically, a quantity of 0.50 mmol (0.17 g) of d(+)‐cellobiose and 0.17 g of catalysts were added into 5 mL of D.I. water containing 6.0 mmol of H_2_O_2_. The reactor was heated at 343 K for 2 h in an oil bath under stirring. After the reaction, the reaction mixture was analyzed by high‐performance liquid chromatography (HPLC; LC‐2000 plus, JASCO) equipped with Aminex HPX‐87H column (flow rate: 0.5 mL min^−1^, eluent: 10 mmol L^−1^ H_2_SO_4_ aq.). Conversion and product yields were calculated by the following equations. Here, *n_i_* is the number of moles of *i* after the reaction, *n*_*i,0*_ is the initial moles before the reaction, and *v_i_* is the number of carbon atoms in species *i*. Others are unknown products that could not be analyzed by HPLC analysis.

$$Conversion\;\left[\%\right]=\;\frac{n_{cellobiose,0}-n_{cellobiose}}{n_{cellobiose}}\times 100$$

$$Yield\;\left[\%\right]=\;\frac{\nu_{i}n_{i}}{n_{cellobiose,0}}\times 100$$

## Conflict of Interests

The authors declare no conflict of interest.

1

## Data Availability

The data that support the findings of this study are available from the corresponding author upon reasonable request.
